# Towards the Instrumentation of Facemasks Used as Personal Protective Equipment for Unobtrusive Breathing Monitoring of Workers

**DOI:** 10.3390/s24175815

**Published:** 2024-09-07

**Authors:** Mariangela Pinnelli, Daniela Lo Presti, Sergio Silvestri, Roberto Setola, Emiliano Schena, Carlo Massaroni

**Affiliations:** 1 Unit of Measurements and Biomedical Instrumentation, Departmental Faculty of Engineering, Università Campus Bio-Medico di Roma, Via Alvaro del Portillo, 21, 00128 Rome, Italy; mariangela.pinnelli@unicampus.it (M.P.); d.lopresti@unicampus.it (D.L.P.); s.silvestri@unicampus.it (S.S.); e.schena@unicampus.it (E.S.); 2 Unit of Automatic Control, Departmental Faculty of Engineering, Università Campus Bio-Medico di Roma, Via Alvaro del Portillo, 21, 00128 Rome, Italy; r.setola@unicampus.it; 3 Fondazione Policlinico Universitario Campus Bio-Medico, Via Alvaro del Portillo, 200, 00128 Rome, Italy

**Keywords:** wearable technology, instrumented facemask, smart module, temperature sensors, personal protective equipment, workers’ safety, breathing monitoring

## Abstract

This study focuses on the integration and validation of a filtering face piece 3 (FFP3) facemask module for monitoring breathing activity in industrial environments. The key objective is to ensure accurate, real-time respiratory rate (RR) monitoring while maintaining workers’ comfort. RR monitoring is conducted through temperature variations detected using temperature sensors tested in two configurations: sensor t_1_, integrated inside the exhalation valve and necessitating structural mask modifications, and sensor t_2_, mounted externally in a 3D-printed structure, thus preserving its certification as a piece of personal protective equipment (PPE). Ten healthy volunteers participated in static and dynamic tests, simulating typical daily life and industrial occupational activities while wearing the breathing activity monitoring module and a chest strap as a reference instrument. These tests were carried out in both indoor and outdoor settings. The results demonstrate comparable mean absolute error (MAE) for t_1_ and t_2_ in both indoor (i.e., 0.31 bpm and 0.34 bpm) and outdoor conditions (i.e., 0.43 bpm and 0.83 bpm). During simulated working activities, both sensors showed consistency with MAE values in static tests and were not influenced by motion artifacts, with more than 97% of RR estimated errors within ±2 bpm. These findings demonstrate the effectiveness of integrating a smart module into protective masks, enhancing occupational health monitoring by providing continuous and precise RR data without requiring additional wearable devices.

## 1. Introduction

In the rapidly evolving landscape of industrial health and safety, monitoring respiratory variables stands as a critical metric for assessing the wellbeing and efficiency of workers exposed to physical, chemical, and other hazards in the workplace [1]. Industrial sectors are characterized by the presence of hazardous substances and strenuous physical demands, potentially impacting respiratory health. The spectrum of respiratory problems includes conditions such as metal fume fever, chronic bronchitis, dry throat, coughing, tightness in the chest, wheezing, and shortness of breath. Workers exposed to welding fumes, for instance, face a 30–40% heightened risk of lung cancer and are more prone to developing respiratory symptoms and experiencing decreased pulmonary functions compared to their unexposed counterparts [1,2,3].

In addition to the inherent respiratory hazards present in industrial settings, demanding workloads further compound the challenges faced by workers. Typical activities in these environments involve prolonged periods of physical exertion, including heavy lifting, repetitive movements, and manual labor tasks. The musculoskeletal strain these activities impose is exacerbated by the consistent use of personal protective equipment (PPE), which adds an additional layer of physical demand [4]. Under Occupational Safety and Health Administration (OSHA) regulations, referring to the standards codified in Title 29 of the Code of Federal Regulations (CFR) 1910 [5], workers in environments lacking sufficient oxygen or containing irritating agents are required to wear respiratory protection devices. This mandate aligns with the health surveillance guidelines for workers exposed to respiratory irritants and toxins [6], ensuring that exposure to hazardous substances does not compromise respiratory health.

The use of PPE, while essential for safeguarding against airborne contaminants, can lead to increased muscular fatigue and discomfort, which can adversely affect both the workers’ health and their productivity [7]. The National Institute for Occupational Safety and Health (NIOSH) [8] provides a checklist for assessing the physical demands and risks of tasks, including monitoring muscular effort and the required range of motion. Additionally, the cognitive load associated with maintaining awareness of one’s surroundings, adhering to safety protocols, and executing tasks with precision further influences workers’ mental faculties. The need for constant vigilance to avoid accidents or exposure to hazardous substances places significant cognitive strain on individuals, further heightening their overall workload.

In this context, monitoring respiratory variables, particularly the respiratory rate (RR), assumes even greater significance, serving as an objective indicator of the physiological strain workers endure. Fluctuations in RR can reflect not only the immediate impact of environmental factors, but also the compounding effects of physical exertion, PPE usage, and cognitive load [9]. Thus, physiological metrics have shown promising results for real-time assessments of physical fatigue, employing respiratory parameters as a quantitative approach to evaluate fatigability during repetitive work [10,11]. The evaluation of both RR and workload can lead to a comprehensive understanding of the physiological demands placed on the workforce. This enables targeted interventions to optimize health, safety, and productivity.

Various technologies are available to users for respiratory monitoring, applicable in clinical and sports fields and daily living activities. The most-used technologies involve direct measurements of the variations in chest circumference caused by respiratory activity by using strain sensors integrated into shirts or belts [12,13,14,15]. RR estimation can also be achieved through electrodes in contact with the chest surface by using photoplethysmography sensors placed at the wrist [16] or in a non-contact manner by using digital cameras [17,18,19]. However, these technologies may be unreliable, especially during work activities, due to motion artifacts. They also necessitate the use of sensors integrated into dedicated wearable systems, which must be worn during work activities alongside traditional PPE. This added weight and restriction in movement can strain the body, contribute to musculoskeletal fatigue, and potentially compromise ergonomic posture. In a wide range of workplace contexts, PPE facemasks are required to be worn as barriers against environmental hazards. Integrating sensors that capture breathing parameters directly into protective masks presents a compelling proposition for seamless and unobtrusive monitoring in industrial contexts. The conceptualization of integrated sensor modules within protective masks represents a paradigm shift in occupational health monitoring, offering a synergistic blend of respiratory protection and real-time data acquisition. The practical benefits of such an integration include reduced encumbrance (since workers are already accustomed to wearing masks), enhanced user compliance, and the reduced influence of motion artifacts during activities.

Different approaches have been tested to capture respiratory patterns with sensors attached to facemasks, mainly via off-the-shelf photoplethysmography (PPG) sensors and pressure sensors integrated within masks [20,21]. Furthermore, recent advancements in sensor technology have introduced innovative solutions such as advanced nanogenerators and flexible sensors based on new nanomaterials [22,23]. These sensors, including nanogenerators utilizing nanofibrous and nanostructured polytetrafluoroethylene (n-PTFE) thin films, have demonstrated success in detecting breathing activities by measuring air-flow-driven pressure changes. As a viable alternative, temperature sensors have been explored as sensing elements to capture breathing parameters [24,25,26,27,28,29,30,31,32] mainly because of response times, low power consumption, form factors, and size. Compared to n-PTFE film sensors, which are typically integrated into the mask’s structure and may affect the mask’s breathability and comfort, temperature sensors require less-invasive modifications, preserving the certification of the mask as a PPE. Polyimide-based humidity sensors, while offering sensitivity to moisture changes which could correlate with RRs, often necessitate complex calibration procedures to remain accurate under different environmental conditions, presenting a challenge for consistent performance in diverse work settings. However, these solutions often entail structural modifications to the facemask or the attachment of sensors within it, which can compromise their certification as PPEs. Consequently, their practical applicability in work scenarios is affected. Some approaches require a complete redesign of the mask, which not only leads to additional costs, but may also fail to be tested in relevant work environments.

To overcome this limitation, in this paper, we propose the design and development of an innovative smart respiratory module that captures breathing patterns through advanced temperature sensors integrated into a facemask. This approach is aimed at providing accurate real-time RR monitoring, enhancing the ability to detect physiological changes and ensuring the system is unobtrusive and comfortable for the user during prolonged use in various industrial environments.

The main aim of this study is to compare the performance of two configurations based on two temperature sensors integrated either inside (t_1_) or outside (t_2_) a facemask via rigid 3D-printed Polylactic acid (PLA) molded structures. The performance of both configurations was assessed by a reference acquisition system, ensuring that they have comparable error rates, thereby providing a reliable choice for integration into the facemask. In addition, the performance of the two configurations (t_1_ and t_2_) was tested under conditions where it is more prone to movement artifacts. This additional analysis will help determine the reliability and accuracy of the thermistor-based module in dynamic industrial environments.

The integration and validation of this module is aimed at providing real-time accurate RR monitoring that could ensure workers’ safety and health in industrial environments without compromising their comfort and productivity. By leveraging the properties of thermistors, the module offers a reliable and non-invasive solution for continuous respiratory monitoring, thereby enhancing the overall effectiveness of occupational health surveillance.

## 2. Materials and Methods

This section details the design and implementation of a respiratory monitoring module integrated into a filtering face piece 3 (FFP3) facemask with an exhalation valve. The module is designed to provide the continuous monitoring of respiratory patterns, thereby enabling the estimation of RR. The development of the module involved several critical stages, each essential for ensuring the reliability and accuracy of the system. These stages included the design of the integration structure to seamlessly incorporate the sensors into the mask without compromising its protective capabilities, and the careful selection and installation of the necessary sensors for precise respiratory parameter detection. Additionally, the module’s development required rigorous testing and validation to confirm its effectiveness under various conditions. Below, we provide a comprehensive overview of the methodologies employed in the module’s design, integration, and validation, highlighting the challenges encountered and the solutions implemented to address them. 

### 2.1. Smart Module Development Process 

**Figure 1:**
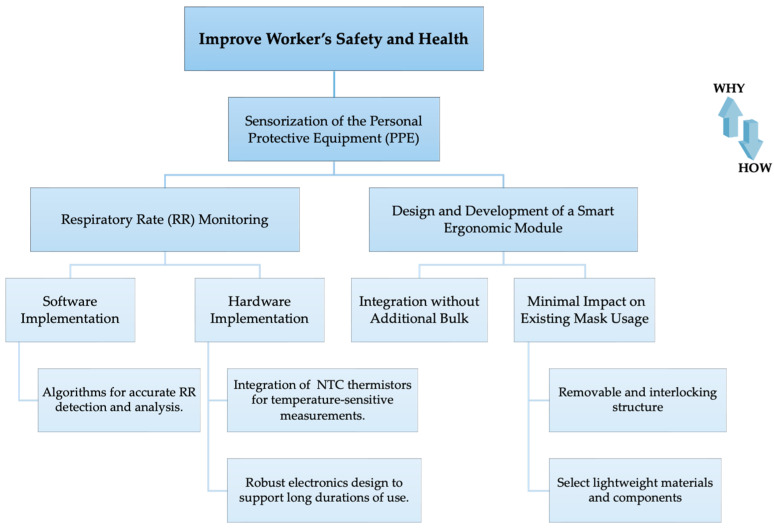
Objective tree diagram.

The device has been engineered both in terms of hardware and software to meet the requirements of respiratory monitoring for workers during their activities. This has been achieved without adding unnecessary bulk, but instead by integrating it directly into the mask they already use. An objective tree of the requirements, along with how and why each was addressed, is detailed in Figure 1.

**Figure 2:**
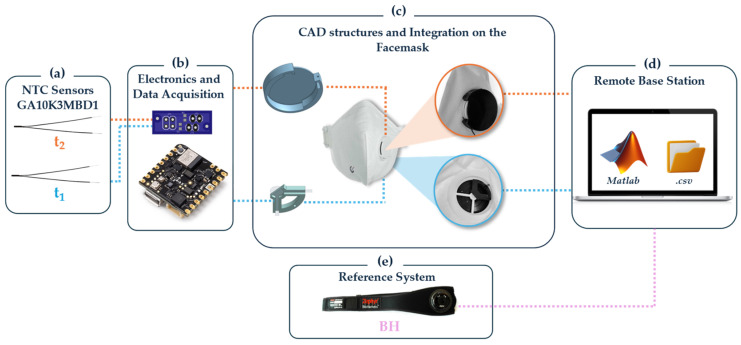
Block diagram of the platform development process and data acquisition mode.

The development process of the system was carefully designed to meet specified requirements and is depicted in Figure 2, detailing the following stages:

- **Sensor Selection and Placement**: Two NTC thermistors (model number GA10K3MBD1, Mini Betacurve by TE Connectivity, Measurement Specialties, Inc. Berwyn, PA, USA, with diameter of 1.1 mm and mass 0.1 g, resistance at 25 °C equal to 10 kΩ ± 0.2%, response time in liquids: 0.4 s, operating temperature range of −40 °C to 125 °C) were chosen. Within the smart module, sensor t_1_ is designed to be placed inside the exhalation valve of the mask, positioned in an area expected to have the highest sensitivity [26], and is less subject to external ambient temperature, thus solely responding to temperature variations related to breath. The drawback of this integration is that it requires drilling into the exhalation valve, which is sealed with a particular material that might raise concerns regarding regulatory compliance. Meanwhile, in the second configuration to be tested, sensor t_2_ is located outside the valve but near the air outlet point, which does not alter the valve structure of the mask, and thus does not require alteration of the mechanical structure of the facemask and its certification.
- **Electronics and Data Acquisition**: The Nicla Sense ME board (Arduino^®^, Monza, Italy) was selected for its compact dimensions (22.86 mm × 22.86 mm) and reduced mass (2 g). It includes data acquisition Bluetooth^®^ 4.2 connectivity, an accelerometer, and a gyroscope.
  To connect the two analog thermistors, t_1_ and t_2_, to the board, a voltage divider circuit is commonly employed. A Printed Circuit Board (PCB) was designed to include two voltage dividers each for the 10 kΩ thermistors (*R_thermistor_*). This configuration was chosen because the 10 kΩ NTC thermistors at 25 °C, when paired with a 10 kΩ resistor (*R_circuit_*) in each voltage divider, create a balanced setup. The output of the voltage divider (*V_out_*) is proportional to the input voltage (*V_in_*), *R_circuit_*, and *R_thermistor_*, as in Equation (1):
  (1) $$V_{out}=V_{in}\cdot \frac{R_{thermistor}}{R_{thermistor\;}+R_{circuit}}$$
  Thus, considering a *V_in_* of 5 V:
  - At 25 °C, where *R_thermistor_* is 10 kΩ (equal to *R_circuit_*), the *V_out_* value is 2.5 V.
  - For NTC thermistors, the resistance decreases with an increase in temperature. Consequently, at temperatures above 25 °C, *R_thermistor_* will be less than 10 kΩ, resulting in *V_out_* increasing beyond 2.5 V. Conversely, at temperatures below 25 °C, *R_thermistor_* will be greater than 10 kΩ, causing *V_out_* to decrease below 2.5 V.
- **Integration of the Smart Module into the Facemask**: Successful integration was facilitated by designing two 3D-printed structures using PLA and the Ultimaker S5 3D printer (Ultimaker, Utrecht, The Netherlands). For thermistor t_1_, an internal quarter-cross-shaped structure was specifically designed to fit into other similar types of valves. This structure has a radius of 13 mm and features a hole with a diameter of 0.65 mm to accommodate the sensor head. For thermistor t_2_ and the Arduino Nicla Sense ME board, an external interlocking structure was crafted. This structure maintains an open exhalation space and includes a passage for t_2_. Above this passage, a slot with a diameter of 39 mm and a 4 mm recess aligned with the exhalation part was created for secure mounting of the electronic board. This design allows for the structure to seamlessly interlock with the valve cover, ensuring efficient placement and stability.
- **Remote Base Station**: Data from the smart module are displayed in real-time within MATLAB to allow for the immediate visualization of waveform shapes, providing crucial feedback on the device’s performance. This real-time data stream is instrumental in verifying the functionality of the sensors and ensuring that they accurately capture the wearer’s breathing pattern.
  In addition to real-time monitoring, the data are also exported to .csv files for subsequent analysis. The data acquisition for this system was intentionally performed using a non-wireless connection. This approach was chosen to ensure a reliable power supply to the board and uninterrupted data acquisition, which is crucial for long-term monitoring and situations where maintaining consistent data integrity and quality are paramount. The non-wireless setup helps to avoid issues related to battery life and wireless connectivity that might introduce gaps or inconsistencies in the data stream.
  Despite using a non-wireless setup for the main data acquisition, the board is equipped with Bluetooth capabilities, offering the flexibility to switch to wireless data transmission where appropriate. This wireless functionality is powered by a 3.7 V Li-Po battery, which includes an integrated battery charger, enhancing the device’s portability and ease of use. Including a rechargeable battery system with Bluetooth connectivity makes the device adaptable to various settings, ranging from clinical to field applications, where mobility and minimal encumbrance are essential. This dual-mode capability ensures that the device can be tailored to meet the specific needs of different research or monitoring scenarios, combining robust and reliable data collection with the flexibility of wireless communication when needed.
- **Selection of the reference system**: The Zephyr BioHarness (by Medtronic Inc., Minneapolis, MN, USA, hereinafter BH) was selected for the reference system, as it is widely recognized for physiological monitoring in industrial settings. It embeds a strain sensor for collecting respiratory signals (sampling rate: 25 Hz) and two dry electrodes for capturing single-lead ECG (sampling rate: 250 Hz). The strain sensors used for respiratory monitoring record the chest wall circumference changes due to respiratory effort, converting mechanical deformation into electrical signals. It was placed on the participants’ chest to record respiratory parameters and compare the data with those obtained from the integrated mask module.

This comprehensive design and implementation plan ensures the smart module’s effectiveness and compliance with the operational and safety standards for industrial use.

### 2.2. System’s Principle of Working 

The proposed system employs an NTC temperature sensor as its sensing element, which is adept at capturing respiratory signals. This sensor operates based on the principle of detecting variations in air temperature associated with breathing activities. The resistance (*R*) of the NTC sensor varies inversely with temperature (*T*), according to the following equation:

(2) $$R\left(T\right)=R_{0}\cdot e^{\beta \cdot \left(\frac{1}{T}-\frac{1}{T_{0}}\right)},$$

where *R*_0_ is the resistance at a reference temperature *T*_0_ (298.15 K), *β* is the Beta Value 3976 K for this sensor, and *T* is the absolute temperature in Kelvin. 

This relationship implies that as the temperature increases, the resistance decreases, adhering to a negative temperature coefficient [33,34]. 

In scenarios where there is no exhaled airflow, such as when the sensor is not being worn or during periods of apnea, the sensor remains exposed to the ambient temperature (T_E_). In these conditions, the output temperature signal and the corresponding resistance signal remain almost constant. On the other hand, during exhalation, the airflow originating from the nose and mouth, typically around 37 °C, interacts with the respiratory module. The dynamics of the sensor in response to varying exhalation temperatures can be outlined as follows:

- If the exhaled T_A_ exceeds the ambient temperature (T_E_), the measured temperature at the respiratory module increases, consequently lowering the resistance.
- Conversely, if T_A_ is below T_E_, the measured temperature decreases, thereby increasing the resistance.
- If T_A_ equals T_E_, both the measured temperature and resistance at the respiratory module remain unchanged.

**Figure 3:**
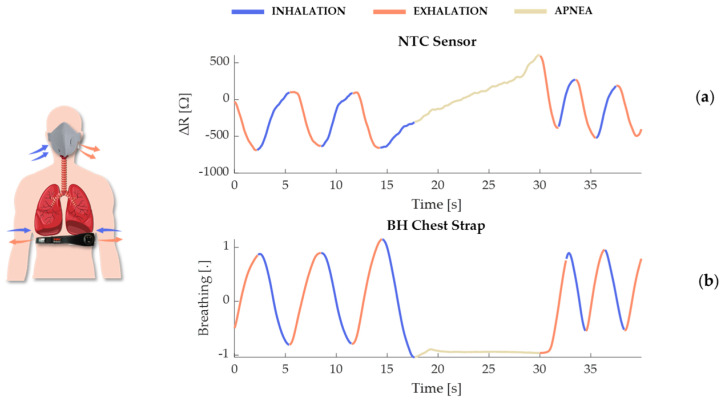
Illustration of the effect of respiration due to air temperature variation on the resistance (ΔR = R(T) − R(T_0_)) of the NTC sensor to be integrated into the facemask (**a**) and on the deformation of the piezoresistive sensor integrated into the BH chest strap (**b**).

The temperature sensors and the BH reference system represent two distinct approaches, each with its own advantages. The temperature sensor, with its high sensitivity to temperature changes, offers powerful detection of temperature changes associated with breathing activity. Its small size and lightweight nature make it ideal for detailed respiratory studies and even clinical monitoring, where even minimal temperature variations are also significant. In contrast, the BH system operates on a piezoresistive sensor principle, where the sensor deforms in response to movements of the rib cage during respiratory phases. This deformation leads to changes in resistance, generating signals indicative of the breathing cycle, with opposing signals observed during inhalation and exhalation (see Figure 3). During apnea, the BH system’s signal remains constant due to the absence of rib cage movement. However, the temperature sensor, while generally stable, can detect slight thermal variations even in the absence of breathing movements. These variations are typically minimal, in the order of Ω, reflecting subtle changes in the microenvironmental temperature around the sensor. As the exhaled warm air is absent during apnea, the sensor gradually adjusts to the ambient temperature, which may account for the observed incremental changes in sensor output. Such sensitivity underscores the temperature sensor’s capability to monitor conditions where even slight physiological or environmental changes are of clinical relevance.

## 3. Feasibility Assessment of the Smart Module

Following the design finalization, the smart module underwent a series of static and dynamic tests to validate the accuracy of t_1_ and t_2_ compared to the reference BH system, particularly under conditions influenced by movement artifacts.

### 3.1. Experimental Setup and Protocol

**Figure 4:**
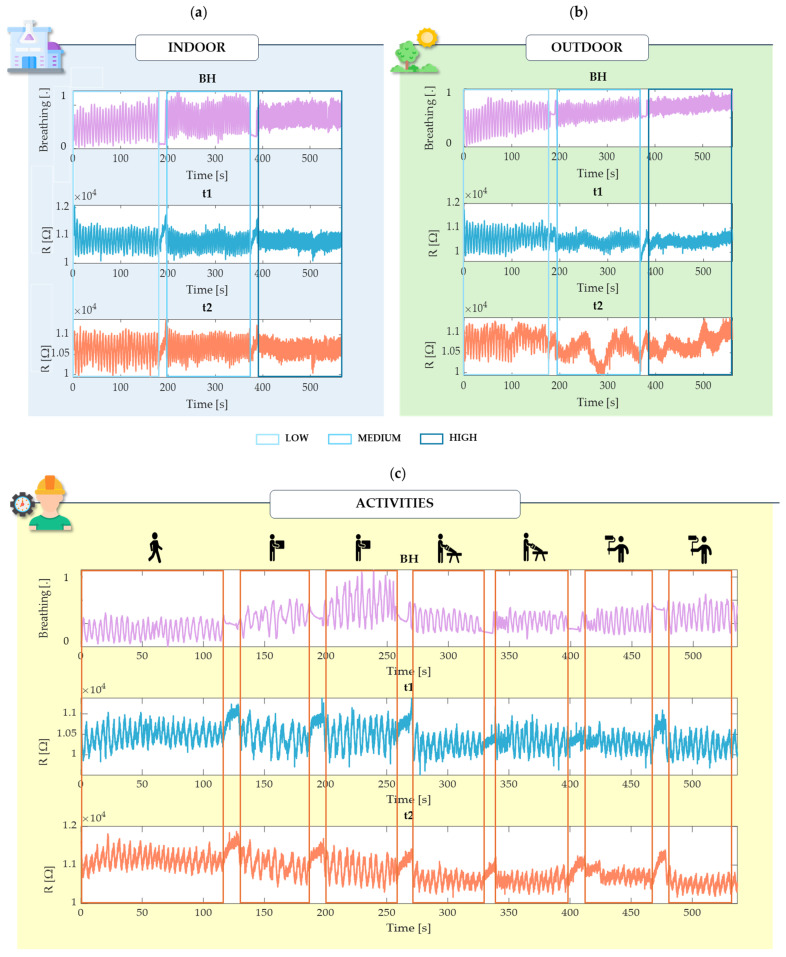
Raw data over time from a single subject (s1) extracted from the BH chest strap and the two thermistors (t_1_ and t_2_) during controlled breathing in indoor (**a**) and outdoor (**b**) environments, and during spontaneous breathing during simulated physical activity (**c**).

Ten healthy volunteers were recruited for this study (seven females and three males, mean ± SD: age 25 ± 6 years, body mass 61 ± 5 kg, height 170 ± 5 cm). Each subject participated in two static tests and one dynamic test defined as follows:

*Static Trials*: These trials were useful to investigate the performance of both configurations of t_1_ and t_2_ compared to the reference in controlled conditions:

- Indoor Environment (Laboratory Environment, approximately 25 °C) (Figure 4a).
- Outdoor Environment (Sun Exposure—approximately 32 °C) (Figure 4b).

In both environments, a simulation of standard breathing activity for 3 min each at guided breathing rates through the App Breathe+ available on the iOS App Store:

○ Low rate: 10 breaths per minute (bpm).
○ Medium rate: 15 bpm.
○ High rate: 30 bpm.

Each environment was tested over a total of 9 min for all ten subjects.

*Dynamic Trial* (Figure 4c): This trial was useful for assessing both configurations under motion artifact conditions. The performance of both t_1_ and t_2_ and the chest strap was evaluated during typical industrial worker activities at self-paced RR, specifically:

- Walking (2 min).
- Lifting Boxes (1 min slow and 1 min fast).
- Cutting (1 min slow and 1 min fast).
- Painting (1 min slow and 1 min fast).

‘Slow’ and ‘fast’ were defined according to the minimum and maximum execution capacities of the subjects.

In each static and dynamic trial, as shown in Figure 4, the different breathing phases—low, medium, and high rates, and also as for the activities—were distinctly separated by 10 s lasting apnea intervals. This structuring was intended to delineate each phase within the testing period clearly. Nevertheless, during these apnea intervals, participants were given the flexibility to slowly release their breath if necessary to ensure their comfort and to account for natural variations in individual breathing patterns, especially between phases of controlled breathing during the static trials.

### 3.2. Data Processing

Raw data collected from the two thermistors and the chest strap were processed in the MATLAB^®^_R2024a environment. The sensors connected to the NICLA Sens board acquired data at an estimated sampling rate of approximately 50 Hz, whereas the BH data had a sampling rate of 25 Hz. To ensure synchronization and facilitate comparison, the signals from the smart module were resampled to 25 Hz, matching the BH’s sampling rate. This resampling was crucial for achieving consistent temporal alignment across all signals.

To isolate low-frequency respiratory components and minimize high-Frequency noise, a Butterworth bandpass filter with cutoff frequencies between 0.01 Hz and 1.0 Hz was applied to all three signals [9]. This filtering step was essential for extracting the relevant respiratory information from the raw data. For each of the static trials and the dynamic trial, the signal was segmented into different segments to provide a more detailed analysis after filtering. In each segment with different characteristics, the RR was then calculated.

**Figure 5:**
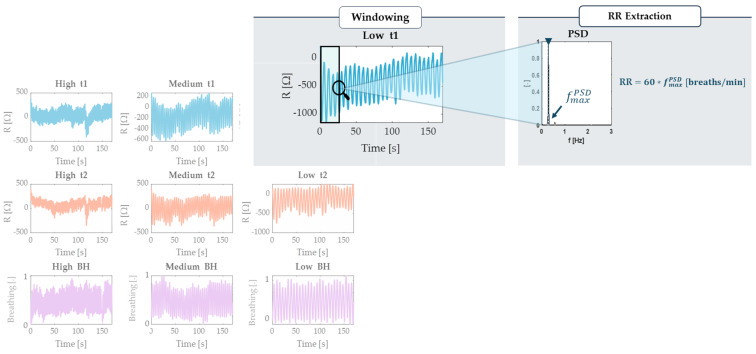
Example of partitioning the signals extracted from BH, t_1_, and t_2_ into the three different controlled frequencies defined for the static trial (in this case, the indoor one). The illustration includes a focus on the analysis process of a signal segment using the Welch method, the selection of a 20 s sliding window and the extraction of the maximum frequency and subsequent RR.

The RR estimation was performed in the Frequency domain, employing the Welch method to calculate the Power Spectral Density (PSD), segmenting the signal into windows of 20 s with a 1 s sliding time (Figure 5). The Welch method is a widely used approach for spectral analysis that reduces the variance in the PSD estimate by averaging periodograms [28,35,36]. In each window, the dominant frequency, representing the point of maximum power, was identified and multiplied by 60 to express the RR in bpm. 

## 4. Results

**Figure 6:**
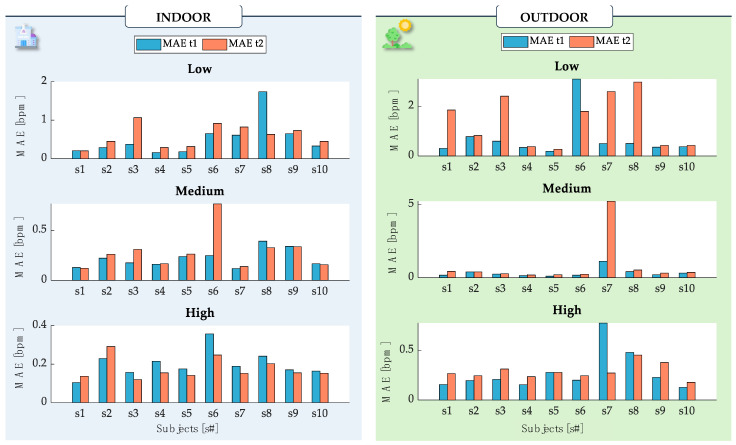
Bar representation of the *MAE* values for t_1_ (light-blue bar) and t_2_ (orange bar) obtained for each of the ten subjects in each of the static trials indoors and outdoors. The *MAE* value was calculated over the entire trial, with further partitioning as described (‘Low’, ‘Medium’, and ‘High’). The *MAE* values reflect the comparison of t_1_ and t_2_ against the BH reference.

To quantify the performance of the proposed system in estimating RR, statistical indices were calculated for each subject and each trial undertaken. Specifically, the Mean Absolute Error (MAE) was calculated separately for sensors t_1_ and t_2_ compared to the BH (Figure 6). MAE was calculated considering the error between the values of each window, as calculated initially, by using the following equation:

(3) $$MAE=\frac{1}{n}\cdot \sum_{i=1}^{n}\left|{RR}_{sensor,i}-{RR}_{reference,i}\right|$$

In addition to MAE, the Mean Absolute Percentage Error (MAPE) was used to provide an overall evaluation across all of the subjects in each trial, reflecting the relative error in percentage terms. The MAPE was calculated using the following formula:

(4) $$MAPE=\frac{100}{n}\cdot \sum_{i=1}^{n}\left|\frac{{RR}_{sensor,i}-{RR}_{reference,i}}{{RR}_{reference,i}}\right|$$

where RR_sensor,i_ is the RR estimated by the temperature sensor (t_1_ and t_2_ separately) and RR_reference,i_ is the RR estimated by the BH signal, with n representing the number of 20 s windows in which the signal was segmented, and i indicating the i-th segment. 

Indoor monitoring MAE showed comparable performances for t_1_ and t_2_. Over all of the ten subjects, MAE values were always lower than 1.74 bpm (t_1_) and 1.06 bpm (t_2_) in low-rate trials (related to the controlled RR at 10 bpm), 0.39 bpm (t_1_) and 0.76 bpm (t_2_) in medium-rate trials (controlled RR at 15 bpm), and 0.36 bpm (t_1_) and 0.29 bpm (t_2_) in high-rate trials (in which RR was controlled at 30 bpm). Complementing the MAE, the MAPE provided additional insights, with t_1_ exhibiting a MAPE of 2.44% and t_2_ showing a MAPE of 2.79% in indoor settings. 

Outdoor monitoring exhibited overall higher MAE values for both the t_1_ and t_2_, with higher MAE especially occurring during low breathing (3.01 bpm, t_1_) and medium breathing due to one volunteer (5.23 bpm, t_2_). High rates during outdoor breathing were comparable with the MAE values obtained in indoor breathing, reaching a maximum value of 0.77 bpm with t_1_. To further quantify the sensor’s performance, the MAPE for outdoor monitoring was 3.36% for t_1_ and significantly higher at 7.62% for t_2_. These figures reflect a greater variability in sensor performance under outdoor conditions, highlighting the challenges posed by environmental factors.

However, it is worth noticing that higher discrepancies between indoor and outdoor breathing were related to slow breathing. In this exercise, in fact, minimal thermal variations caused by the environment and the different dynamics of the instruments may introduce potential sources of noise in the signals and cause slightly different values of estimated RR, as previously observed in [24].

We aggregated the RR across all ten subjects to better investigate the overall performances in the static trials. Those data were used in a Bland–Altman analysis and error distribution calculation. The Bland–Altman analysis provides two primary metrics: the Mean of Differences (MOD) and the Limits of Agreement (LOA) [37]. 

MOD reflects the average deviation between the two instruments’ values, while LOA defines the range within which approximately 95% of data points should fall. This method involves plotting the difference between the two measurements against their average. These metrics were calculated as follows:

(5) $$MOD=\frac{1}{n}\sum_{i=1}^{n}\left({RR}_{sensor,i}-{RR}_{reference,i}\right)$$

(6) LOA=MOD ±1.96×SD

where SD is the standard deviation of the differences between the two measurements. This investigation considered all of the subjects’ RR data across each trial, combining each phase and leading to the presentation of individual Bland–Altman plots.

To obtain the error distribution, the error ΔRR_i_ was calculated as RR_sensor,i_ − RR_reference,i_ by considering all of the subjects’ RR data. The cumulative distribution function (CDF) was employed to analyze these errors with a resolution of 1 bpm.

**Figure 7:**
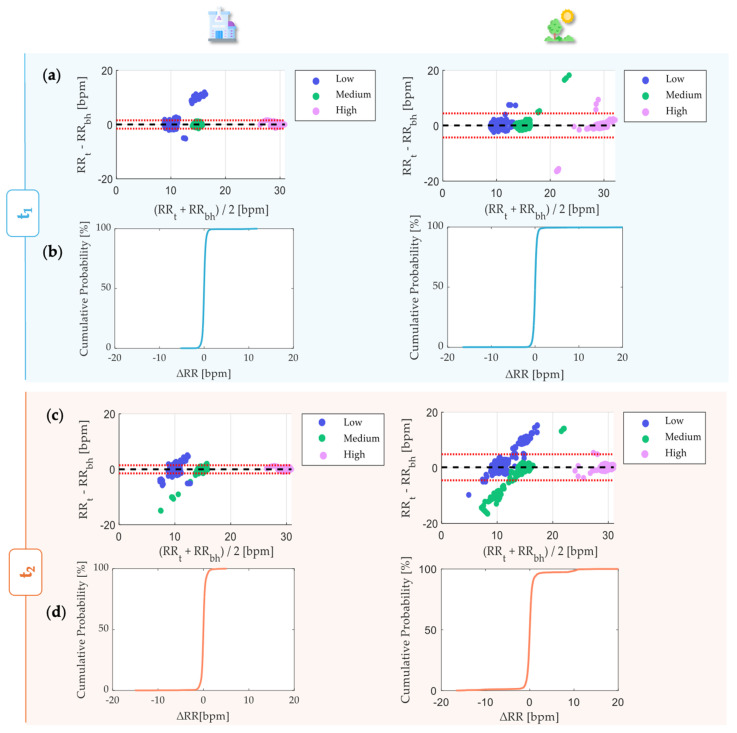
Bland–Altman analysis for the static trials for t_1_ (**a**) and t_2_ (**c**). Alongside each trial, the corresponding CDF is displayed, demonstrating the acceptability of the measurements for t_1_ (**b**) and t_2_ (**d**). MOD is reported with a black dashed line, while LOAs are reported as red dashed lines.

The Bland-Atman plots (Figure 7a,c) evidenced MOD approximately equal to 0 in both of the scenarios (indoor and outdoor) for both of the sensors (t_1_ and t_2_). Each Bland–Altman plot included 4800 RR values. Indoor monitoring with t_1_ allowed us to estimate the RR with LOAs equal to ±1.54 bpm comparable to RR estimations performed via the t_2_ in the same condition (LOA = ±1.46 bpm) in the RR range between 8 and 31 bpm. Both the t_1_ and t_2_ exhibited higher LOAs in the case of outdoor monitoring, especially LOAs = ±4.34 bpm for t_1_ and ±4.68 bpm for t_2_, approximately three times higher than indoor-monitoring LOAs. The CDF results indicate that 99.62% and 99.18% of the RR values are within ±2 bpm with respect to the referenced RR in indoor monitoring using t_1_ and t_2_. Although t_1_ exhibited similar results (99.42%) in outdoor monitoring, t_2_ showed a slightly lower percentage of RR (96.70%) within ±2 bpm in this context (Figure 7b,d). 

The reported table below, Table 1, summarizes the Bland–Altman and distribution values for t_1_ and t_2_ during outdoor and indoor tests.

Furthermore, the dynamic trials were specifically designed around the concept of repeated activities, as excessive repetition of movements has been shown to increase risk indices and cause accelerated breathing, according to the NIOSH checklist. These activities included walking, lifting boxes, cutting, and painting, with variations in speed to mimic the range of work intensities encountered in industrial settings. 

The MAE proved useful for conducting inter-subject analyses and identifying how raw data collected from the BH exhibited marker motion artifacts, a problem already evidenced in the Introduction. It was apparent that the chest strap, which is influenced by torso movements, often showed greater fluctuations. This observation suggests that considering torso movement can lead to a more nuanced understanding of error rates, potentially lowering them when considering these dynamics.

**Figure 8:**
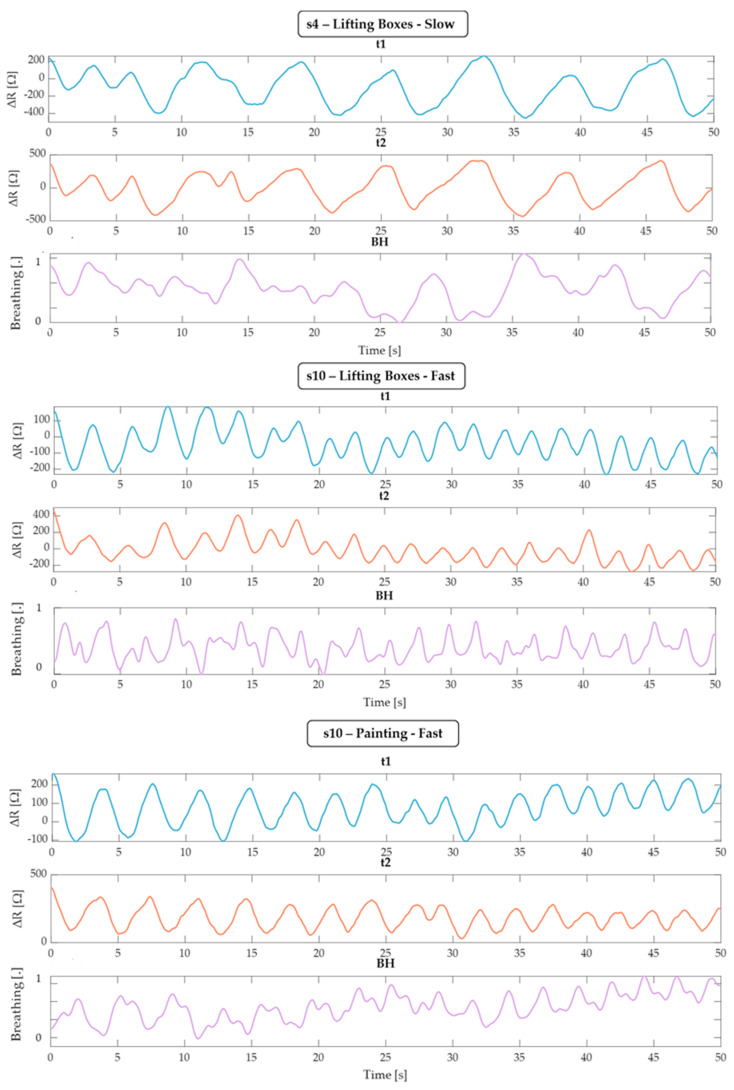
Representation of a 50 s window of the t_1_, t_2_, and BH signals relating to subjects s4 and s10, which shows an evident movement pattern for the BH band, while the respiratory cycles of the sensors integrated in the mask are evident.

To perform an unbiased comparison between t_1_, t_2_, and BH, we priorly visually checked all of the raw data provided by BH and excluded three tests (subject 4 being slow when lifting boxes, subject 10 lifting boxes fast and painting fast). Figure 8 clearly shows the consistent and coherent trends observed in the data from t_1_ and t_2_, whereas the BH data are noticeably affected by motion artifacts. This discrepancy highlights the reliability of the integrated thermistor sensors (t_1_ and t_2_) in accurately capturing respiratory patterns, even in the presence of movement, compared to the more artifact-prone BH chest strap. All of the other tests were used to calculate the MAE and error distribution, and a Bland–Altman test was performed for the static tests.

**Figure 9:**
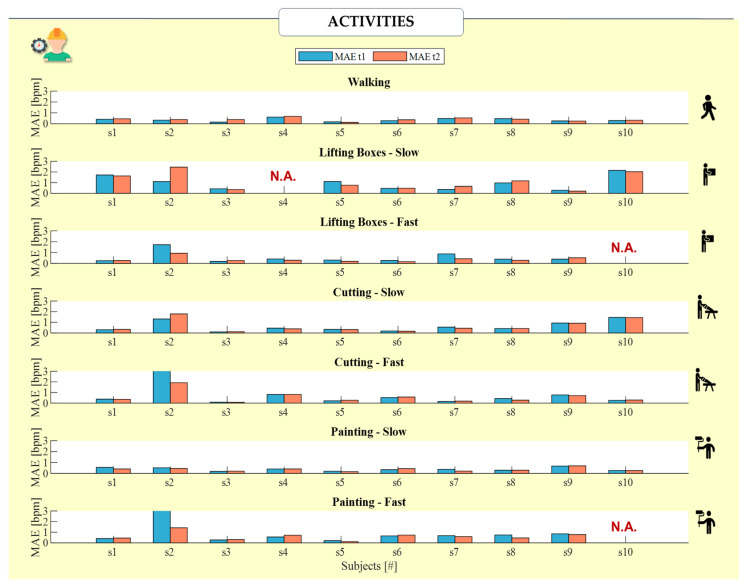
Bar representation of the MAE values for t_1_ (light-blue bar) and t_2_ (orange bar) obtained for each of the ten subjects (s1–s10) in each of the partitioned dynamic trials. The values exceeding 3 bpm due to BH influenced by motion have been excluded from the analysis (N.A.).

Figure 9 reports the MAE calculated for each subject during all of the dynamic activities. In the majority of subjects, excluding those in which the refences system’s signal is influenced by the artifacts, the MAE is in line with the results obtained in static trials. Moreover, comparable MAEs have been obtained for t_1_ and t_2_ in most cases, with maximum differences between t_1_ and t_2_ being 3 bpm for subject #2 during fast painting and cutting.

Additionally, the MAPE for each sensor, t_1_ and t_2_, was calculated across all of the subjects, but was broken down by individual activity to provide a nuanced view of sensor performance under dynamic conditions. These MAPE values are reported in Table 2, offering a comprehensive overview of sensor accuracy during specific physical activities.

**Figure 10:**
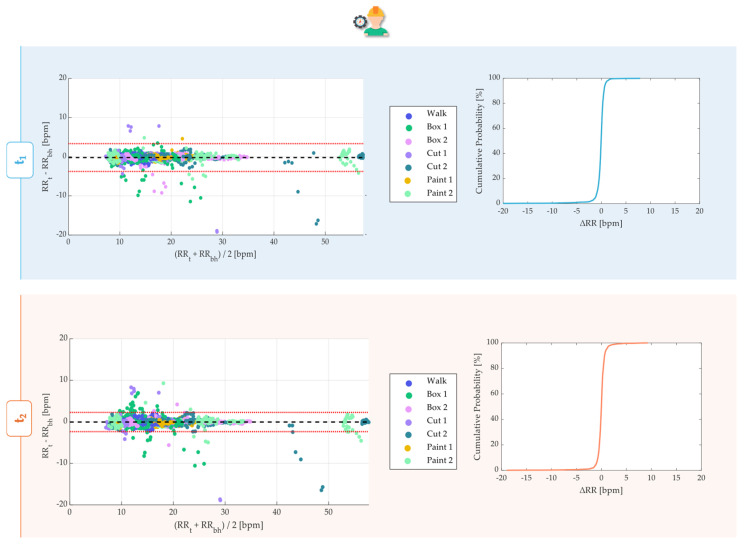
Bland–Altman analysis for the dynamic trial for t_1_ and t_2_, representing the data of all ten subjects in each of the seven activities. In the legend, ‘1’ stands for the activity executed slowly and ‘2’ stands for a faster execution as described in the experimental protocol. Alongside each plot, the corresponding CDF over 4620 values is displayed. MOD is reported with a black dashed line, while LOAs are reported as red dashed lines.

As far as the Bland–Altman analysis is concerned, except for the data subject to artifacts, the performance of the sensors was consistent with that obtained for the statics (Figure 10). The distribution of data within the plot for each sensor consistently showed the groupings corresponding to higher frequencies for tests that required greater effort (‘Fast’) and lower (‘Slow’) effort.

In the face of a greater variability of the free RRs performed by the subjects, the values of the MODs approximately equaled 0 for both t_1_ and t_2_. While t_1_ allowed us to estimate the RR with a LOA range equal to ±3.55 bpm, t_2_ allowed for comparable RR estimations performed in the same conditions within a range of ±2.30 bpm. Both the t_1_ and t_2_ exhibited higher LOAs than the indoor monitoring seen before, but lower than the outdoor ones. The CDF analyses revealed that 97.54% and 97.08% of RR values were within ±2 bpm with respect to the referenced RRs, respectively, with t_1_ and t_2_.

In order to sum up the MOD, LOAs, and percentage of detected values, all of this information is grouped into Table 3 below.

Thus, the temperature sensors demonstrated high reliability in detecting higher respiratory frequencies. This reliability was consistent even during dynamic activities, further proving the robustness of the system.

## 5. Discussion and Conclusions

The core objective of this study was to assess the effectiveness of a respiratory monitoring module integrated into a mask—a tool already mandatory for the targeted workforce. This integration not only minimizes the bulkiness of additional equipment, but also enhances the ease of detecting breath, which is crucial for monitoring worker fatigue and the onset of physical issues.

Despite initial expectations, this study reveals that the performances of the internal temperature sensor t_1_ and the external t_2_ were largely comparable and reliable across various conditions—indoors, outdoors, and during active movements—when validated against the BH reference system. Placing the sensor inside the mask directly in the path of exhaled air ensures the immediate and accurate detection of respiratory activity. The temperature sensor captures the warm air from exhalation, leading to a rapid decrease in resistance and a clear signal of breathing. This was evident in our results, where the internal sensor t_1_ consistently produced low average errors, aligning with findings from the literature [24]. Although it is slightly more accurate, t_1_ requires modifications for certification, which may complicate its integration into existing safety frameworks. 

On the other hand, t_2_—despite being more susceptible to external environmental variations, such as environmental temperature fluctuations, which makes it more variable during data acquisition phases—has shown promising results that are nearly on par with t_1_. This makes t_2_ a viable option since it does not necessitate the same level of certification modifications as t_1_, potentially easing the integration process within current regulatory and operational constraints. Thus, these findings suggest that while t_1_ might offer slightly superior accuracy, the ease of integration and lower regulatory hurdles associated with t_2_ make it an equally compelling choice for practical applications.

Moreover, both sensors demonstrated robust performance during dynamic trials, which involved repetitive movements known to increase risk indices and accelerate breathing. In fact, they not only showed comparable results for both t_1_ and t_2_ with an acceptability threshold exceeding 97.1%, but they also illuminated the path toward an innovative solution that addresses challenges faced by traditional measurement systems like the BH chest strap. Unlike the BH system, which could be susceptible to movement artifacts due to its placement on the torso, the integrated sensors in our study demonstrate a remarkable resilience to such disturbances; by positioning sensors closer to the point of respiratory activity and securely within the mask, our module minimizes the influence of external movements that typically affect torso-mounted devices.

Our study further provides valuable insights into the utility of MAE for conducting inter-subject analysis and determining how the respiratory monitoring system responds to various breathing patterns. All participants used both nasal and oral breathing during the tests, and the errors recorded were consistently within acceptable limits, affirming the system’s accuracy and reliability. Notably, the error remained impressively low even during dynamic activities where movement could potentially disrupt sensor readings.

The Bland–Altman plots further elucidated that most errors were associated with lower breathing frequencies, observable in both indoor and outdoor environments across both sensors. Nevertheless, the sensors exhibited exceptional reliability in capturing higher respiratory frequencies. This consistent performance persisted even during physically intensive tasks, underscoring the robustness of our system. Additionally, CDF analyses effectively illustrated that the error was tightly concentrated around ±2 breaths per minute, reinforcing confidence in the system’s precision. These results, together with the MOD and LOAs, have been comprehensively documented in Table 1 and Table 2.

Looking ahead, this study opens several pathways for further development. There is potential to leverage the full suite of sensors on the Nicla Sense ME board, including accelerometers, to more accurately assess the physical dynamics of workers, such as head rotations and body axis movements. Furthermore, future iterations of the prototype could explore combining these sensors to enhance data richness and precision. Such developments may include modifications to the circuitry for signal conversion and the potential addition of an amplifier to improve signal strength and quality.

Moreover, expanding the use of similar modules in other types of PPE could provide comprehensive safety and health monitoring solutions across different industrial sectors. Another promising area of research involves correlating the intensity of physical activities with RRs to more effectively gauge the risk index defined by NIOSH, especially concerning repetitive movements. Finally, recognizing the feedback regarding testing settings, future expansions of the research will critically analyze the influence of environmental parameters such as temperature, humidity, wind, and dust under extreme working conditions. This analysis will validate the sensor performance in environments experiencing extreme temperatures ranging from as high as 40 °C to as low as those observed during harsh winters. Variations in humidity and wind can significantly alter sensor sensitivity and readings, requiring calibration efforts and the development of algorithms to distinguish between respiratory-induced changes and environmental effects. Additionally, dust can impair sensor function, necessitating protective measures such as specialized casings with filters to maintain sensor integrity. Understanding and mitigating these influences will enhance workplace safety protocols and expand the sensor applicability across diverse and extreme industrial scenarios.

Understanding the relationship between increased RRs and higher risk due to frequent repetitions could significantly enhance workplace safety protocols, making them more responsive to the physiological impacts of specific tasks. Future developments in this research will aim at significantly extending the participant demographic, encompassing a broader age range and including individuals with varying levels of respiratory fitness, particularly those facing respiratory challenges. Such expansion is critical for assessing the performance of the sensor system across a diverse user base and for evaluating its effectiveness and adaptability in real-world scenarios. The scope of testing durations and conditions could be expanded to capture a wider array of respiratory patterns and physiological responses.

This exploration into integrated respiratory monitoring systems underscores the significant benefits and potential improvements for occupational health practices. The comparability of t_1_ and t_2_, along with their respective advantages and limitations, highlights the importance of sensor choice based on specific application needs and regulatory considerations, thereby enhancing workers’ safety and wellbeing in diverse industrial environments.

## Figures and Tables

**Table 1:** MOD, LOAs, and percentage of acceptability of sensors t_1_ and t_2_ in each phase.

	Sensor t_1_		Sensor t_2_	
	INDOOR	OUTDOOR	INDOOR	OUTDOOR
MOD [bpm]	0.03	0.11	−0.01	0.17
LOA1 [bpm]	+1.57	+4.45	+1.45	+4.86
LOA2 [bpm]	−1.51	−4.24	−1.47	−4.51
% of RR within ±2 bpm [%]	99.6	99.4	99.2	96.7

**Table 2:** MAPE values for each activity performed by all ten subjects.

	MAPE [%]	
Activities	Sensor t_1_	Sensor t_2_
WALKING	2.49	2.76
LIFTING BOXES—SLOW	6.46	7.24
LIFTING BOXES—FAST	2.93	2.22
CUTTING—SLOW	4.25	4.68
CUTTING—FAST	2.86	2.61
PAINTING—SLOW	2.53	2.41
PAINTING—FAST	3.84	3.26

**Table 3:** MOD, LOAs, and percentage of acceptability of sensors t_1_ and t_2_ considering all of the dynamic tests.

	Sensor t_1_	Sensor t_2_
	ACTIVITY	ACTIVITY
MOD [bpm]	−0.21	−0.05
LOA1 [bpm]	+3.34	+2.25
LOA2 [bpm]	−3.76	−2.36
% of RR within ±2 bpm [%]	97.54	97.08

## Data Availability

The data presented in this study are available upon request from the corresponding author. The data are not publicly available due to privacy reasons.
